# Augmenting intraoperative ultrasound with preoperative magnetic resonance planning models for percutaneous renal access

**DOI:** 10.1186/1475-925X-11-60

**Published:** 2012-08-24

**Authors:** Zhi-Cheng Li, Kai Li, Hai-Lun Zhan, Ken Chen, Jia Gu, Lei Wang

**Affiliations:** 1 Shenzhen Key Laboratory for Low-cost Healthcare, Key Lab for Health Informatics, Shenzhen Institutes of Advanced Technology, Chinese Academy of Sciences, Xueyuan Avenue 1068, Shenzhen, 518055, China; 2 Department of Medical Ultrasonics, The Third Affiliated Hospital of Sun Yat-Sen University, Guangzhou, 510630, China; 3 Department of Urology, The Third Affiliated Hospital of Sun Yat-sen University, Guangzhou, 510630, China

## Abstract

**Background:**

Ultrasound (US) is a commonly-used intraoperative imaging modality for guiding percutaneous renal access (PRA). However, the anatomy identification and target localization abilities of the US imaging are limited. This paper evaluates the feasibility and efficiency of a proposed image-guided PRA by augmenting the intraoperative US with preoperative magnetic resonance (MR) planning models.

**Methods:**

First, a preoperative surgical planning approach is presented to define an optimal needle trajectory using MR volume data. Then, a MR to US registration is proposed to transfer the preoperative planning into the intraoperative context. The proposed registration makes use of orthogonal US slices to avoid local minima while reduce processing time. During the registration, a respiratory gating method is used to minimize the impact of kidney deformation. By augmenting the intraoperative US with preoperative MR models and a virtual needle, a visual guidance is provided to guarantee the correct execution of the surgical planning. The accuracy, robustness and processing time of the proposed registration were evaluated by four urologists on human data from four volunteers. Furthermore, the PRA experiments were performed by the same four urologists on a kidney phantom. The puncture accuracy in terms of the needle-target distance was measured, while the perceptual quality in using the proposed image guidance was evaluated according to custom scoring method.

**Results:**

The mean registration accuracy in terms of the root mean square (RMS) target registration error (TRE) is 3.53 mm. The RMS distance from the registered feature points to their average is 0.81 mm. The mean operating time of the registration is 6'4". In the phantom evaluation, the mean needle-target distance is 2.08 mm for the left lesion and 1.85 mm for the right one. The mean duration for all phantom PRA tests was 4'26". According to the custom scoring method, the mean scores of the Intervention Improvement, Workflow Impact, and Clinical Relevance were 4.0, 3.3 and 3.9 respectively.

**Conclusions:**

The presented image guidance is feasible and promising for PRA procedure. With careful setup it can be efficient for overcoming the limitation of current US-guided PRA.

## Background

In minimally invasive urology, optimal outcomes of several interventional surgeries such as Percutaneous Nephrolithotomy (PCNL) and tumor radiofrequency ablation (RFA) highly depend on a successful percutaneous renal access (PRA) [1]. PRA is a difficult procedure, requiring an accurate, safe and rapid needle puncture from skin to an intrarenal target.

Intraoperative image guidance is crucial for a successful PRA, because needle puncture without image guidance is imprecise and an injury to vital structure could take place [2]. Traditionally, the PRA is performed under the X-ray guidance [3]. The main disadvantage is the radiation exposure for patient and medical personnel especially. The ultrasound (US) has been proven to be a good alternative, as it is radiation-free, real-time and easy-to-use and [4]. However, the abdominal US is often related to limited anatomy identification and targeting abilities, providing only two-dimensional (2D) anatomical information with poor quality. Furthermore, the US guided PRA may become difficult or even impossible when the target lesion is sonographically obscure or surrounded by confounding tissues [5]. Other imaging modalities for guiding PRA such as C-arm system and intraoperative computer tomography (CT) were also reported [3]. The main drawbacks are the harmful radiation exposure and high deployment cost. Currently, new image guidance for PRA with explicit anatomy identification and accurate target localization is still on demand.

One promising solution is to augment real-time US images with preoperative image modalities such as CT or magnetic resonance (MR) [6]. This technique (1) allows surgeons to calculate an optimal puncture trajectory preoperatively with the 3D reconstructed CT or MR, and (2) provides surgeons with more accurate targeting ability and more intuitive 3D guidance. Urologists have found that a puncture trajectory planning can significantly facilitate an accurate and safe PRA [7]. A major challenge here is to register the preoperative images and the real time US [6]. After such a registration, the spatial correspondence between pre- and intraoperative image modalities can be known. The preoperative planning models can then be transferred onto the *in situ* patient in the operating room (OR).

When registration technique is used for the purpose of PRA, it requires the registration to be accurate, robust and easy to manipulate, considering the kidney shift and deformation due to respiratory motion. Existing feature-based registration methods such as the volume navigation (VNav) method used in commercial ultrasound machine often suffer from inaccuracy, mainly because it uses less efficient registration landmarks and does not take the breathing motion into account [8,9]. On the other hand, the image intensity-based registration methods were reported to be less reliable, as the intensity correlation between different modalities is not that explicit [10,11].

This paper evaluates the feasibility and efficiency of a proposed augmented US-based image guidance framework for PRA. A key consideration in designing this framework is the acceptability and feasibility in clinical environment. The major features of the proposed framework are: 1) A preoperative surgical planning approach is presented to define an optimal needle trajectory with 3D kidney surface and vascular structures extracted from the MR volume; 2) An accurate and robust MR to US rigid registration using orthogonal geometry is proposed to transfer the preoperative planning into the intraoperative context, where the breathing motion is tracked to minimize the impact of kidney deformation; 3) A 3D visual guidance is implemented by augmenting the intraoperative US with preoperative MR planning models and a virtual needle, in order to aid the correct execution of the planning. Finally, the system is evaluated in two stages. First, registration performance in terms of accuracy, precision and processing time are measured on human data from four volunteers. Second, four urologists are asked to perform the image-guided PRA on a kidney phantom. Both puncture accuracy and perceptual quality in using the presented image guidance are evaluated.

The rest of the paper is organized as follows. Section 2 presents the image guidance framework in details, including the preoperative planning and the intraoperative guidance. Section 3 describes the experimental studies and related evaluation results and discussions. Section 4 concludes this paper.

## Methods

### Overview of the proposed PRA workflow

The proposed augmented ultrasound-based image-guided framework comprises a diagnostic ultrasound device, an optical tracker with reflective markers, and a main computer. The proposed image-guided surgical workflow is as follows.

**Figure 1:**
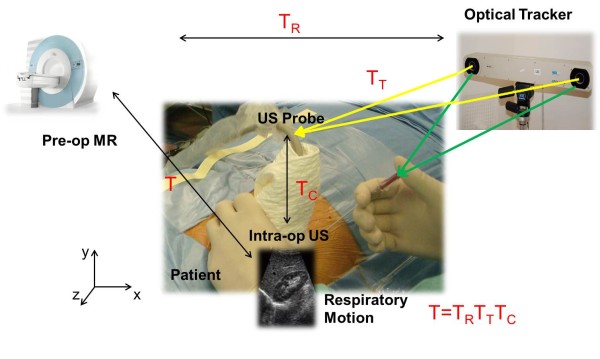
Overview of the proposed image-guided renal intervention.

First, an MR scanning is performed on the patient, where the vessels exhibit high contrast relative to their background. The kidney, vessels, and skin are then extracted from the MR volume as a 3D model. Then surgeons can preoperatively define a needle trajectory that avoids vital vessels and facilitates an effective treatment. During the surgery, the US slices of the kidney are acquired at the maximum exhalation positions of each respiratory circle. The preoperative data and the surgical planning are then registered to the calibrated US images using two pairs of orthogonal slices, such that the planning can be transferred into the OR. The position of the needle tip is read by the optical tracker in real time. By augmenting the US with the MR models and a virtual needle, a 3D visual guidance is provided to facilitate the hand-eye coordination of the treating surgeon, such that the PRA can be performed accordance with the planning. The overview of the entire framework is shown in Figure 1. Next, we will describe in details the proposed framework.

### The MR-based preoperative planning

**Figure 2:**
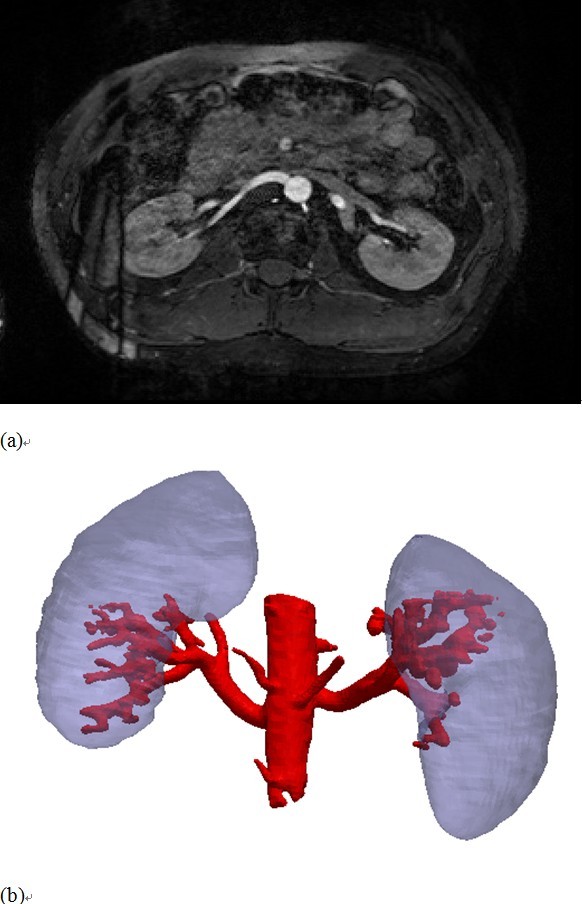
**The preoperative surgical planning using MR data. (a)** The true-FISP MR abdominal images. The vessels exhibit relatively high contrast. **(b)** 3D visualization of the extracted kidney and vessels as geometric models. For clarity, the skin is not shown.

In this subsection, we present a 3D visualized environment that combines convincing virtual representation of the kidney surface and the renal vascular structures from preoperative MR, allowing surgeons to plan an optimal needle trajectory. Here “optimal” means suitable and safe for the interventional puncture. The precondition of such a planning is to obtain abdominal volumetric MR images with high contrast and high spatial resolution and then to extract the 3D geometric description of the kidney and renal vessels. Thusly, we use the True Fast Image with Steady-state Precession (true-FISP) MR sequence [12] to acquire the volume data, as shown in Figure 2a. Note that other contrast-enhanced imaging modalities such as MRA (Magnetic Resonance Angiography) are also suitable for the planning.

Due to the high contrast relative to the background, here we segment the vascular structures using a neighbourhood connected region growing algorithm for the sake of less manual interaction. Specifically, the algorithm starts with placing one or two seeds in the vessel region and incrementally segments the vessels by recruiting neighbouring voxels according to an intensity threshold [13]. As the contrast-to-noise ratio of the kidney is not that high, the kidney is segmented in a manual way. After manually segmenting the skin, all segmented models are then smoothed using a 3D Gaussian kernel and converted into triangulated meshes by means of the marching cubes algorithm [14]. Then, the triangulated meshes, including vessels, kidney, and skin, are fused and merged into one geometric model, as shown in Figure 2b. Based on such a visualized anatomy, a needle trajectory planning that is optimal for the PRA can be defined as an entry point on the skin and a target point in the kidney. Generally, the needle entry on the skin is near the 11th intercostal space, while the trajectory should avoid all large vessels.

### The augmented US-based intraoperative guidance

We expect to provide intraoperative guidance by transferring the preoperative planning to the intraoperative conditions and augmenting the US with preoperative anatomical models. The critical problem here is to register the MR volume to the US slices considering the patient respiration. This problem can be formulated as follows:

Let *x*_US,*i*_ be a pixel position in the intraoperative US plane while *x*_MR,*j*_ be a voxel position within the preoperative MR volume. Given two point sets *X*_US_ = {*x*_US,*i*_} and *X*_MR_ = {*x*_MR,*j*_}, *i =* 1,…, *N*, *j =* 1,…, *M*, we aim to find a transformation *T*_*R*_ that represents the spatial correspondence from the preoperative MR volume *S*_MR_ to the intraoperative space *S*_tra_ defined by the tracker. Based on *T*_*R*_, the preoperative models such as the planning and the 3D geometric anatomy can be transferred into the intraoperative space. Therefore, the puncture can be guided to be coincident with the planning. Next, we will describe the navigated intervention in detail.

#### (i). US calibration

The intraoperative processing starts with the US calibration that maps the pixel position set *X*_US_ to the intraoperative space *S*_tra_. In particular, we first determine a homogeneous transformation *T*_*C*_ that maps pixel positions from the 2D US slices to the local 3D space defined by the optically-tracked markers mounted on the US probe. Let *y*_US,*i*_ denote the location of pixel *x*_US,*i*_ in the local probe space, then we have

(1) $$y_{US,i}=T_{C}\cdot x_{US,i}$$

After a further transformation *T*_*T*_ from the probe space to the tracker space, all US slices can be localized in the same space *S*_tra_ as

(2) $$z_{\text{US},i}=T_{T}\cdot y_{\text{US},i}$$

The calculation of *T*_*C*_ and *T*_*T*_ will be given later in Section 3. After the calibration, each pixel in all acquired US slices can be positioned in the intraoperative space as *Z*_US_ = {*z*_US,*i*_}.

#### (ii). Respiratory gating

Because of the organ motion due to respiration, inconsistency will occur between the positions of the anatomical features such as vascular structures in different US slices. This will lead to significant registration error. Therefore, we expect to acquire US slices at the same stages of the respiration cycles. It has been shown that for free respiration the kidney assumes the same positions at equivalent lung volumes [15]. Moreover, the end-exhale represents the longest natural pause in a cycle [16]. Thus, we expect to use only US slices at the maximum exhalation positions.

**Figure 3:**
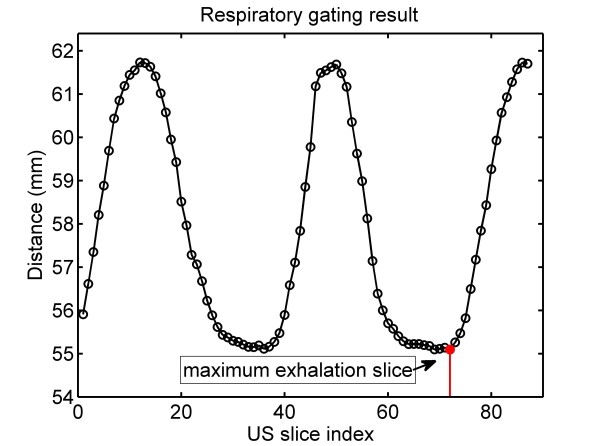
**The respiratory gating result.** The distance from marker centroids to reference axis l0 is shown. The hollow circles denote the US slices acquiring. 87 slices are acquired at a rate of 20 slices per second. As for this group of slices, the 72nd slice that corresponds to the minimum distance is selected as the maximum exhalation US slice, as shown by the red circle.

This can be achieved by an optical tracking based respiratory gating technique. First, the US probe is placed on the caudal end of the patient’s sternum, where the US slice plane is approximately parallel to the transverse section of the human body. At this position, we define a cranio-caudal line *l*_0_ that is perpendicular to the transverse section of the human body and passes through the central point of the US transducer face. The line *l*_0_ is considered as a reference axis. In order to acquire one US slice of interest at the maximum exhalation, the probe is placed at the specified location, and a certain number of slices are acquired and stored at a stable acquiring rate. Meanwhile, the instantaneous distance from the centroid of the optically-tracked markers mounted on the probe to the reference axis *l*_0_ is calculated. The US slice with the minimum distance is considered as the required maximum exhalation slice, as shown in Figure 3. In such a case, the maximum exhalation serves as a trigger for slice selection, accounting for the breathing motion and ensuring consistency of all used US slices.

#### (iii). MR to US affine registration

We aim to transfer the preoperative planning onto the patient *in situ* by means of MR to US registration. With respect to clinical demand, surgeons prefer an effective image guidance that optimizes the surgeon's hand-eye coordination, but are often reluctant to accept over complicated workflow. To this end, we do not use a MR to densely-sampled US volume registration studied by several groups, which requires overlong intraoperative processing time [17]. Meanwhile, we decide not to use the VNav method after careful consideration, which often suffer from less accuracy [8]. Instead, we propose an efficient approach on top of an affine registration based on two pairs of orthogonal US images.

The affine registration is equivalent to find a transformation *T*_*R*_ that best aligns the target point set *X*_MR_ with the source point set *Z*_US_. The iterate closest point (ICP) algorithm [18] is often used to estimate a transformation that minimizes the mean square distance between two point sets. However, the original ICP is sensitive to the initial pose and could be prone to fall into local minima, especially for noisy feature sets, e. g., a point-cloud selected in US images. Moreover, the free landmark extraction is often inefficient and leads to inaccurate registration, mainly because it utilizes insufficient “geometrical information”. In order to overcome these limitations, we propose an orthogonal-slice ICP (OICP) algorithm detailed as follows.

First, the US probe is swept near the 11th intercostal space. A few US images are acquired at the maximum exhalation positions based on the respiratory gating described above. Two pairs of almost orthogonal images, *U*_1_, *U*_2_, *U*_3_ and *U*_4_ are selected, where *U*_1_, *U*_2_ are approximately parallel to the transverse section of the body and *U*_3_, *U*_4_ are acquired with the probe along the midaxillary line. *U*_1_, *U*_2_ contain clearly visible hilum vein, inferior vena cava (IVC), and a transverse kidney contour while *U*_3_, *U*_4_ contain a clearly visible longitudinal kidney contour. Note that both the transverse and longitudinal contours should better be complete, or at least represent most of the kidney.

The kidney surface and large vessel surface are used as registration features for the best alignment of the US slices and the MR volume. The target features *X*_MR_ consist of kidney surface denoted as *K*_MR_ = {*k*_MR,*j*_}, *j =* 1,… *K*_1_, and vessels including hilum vein surface and IVC surface denoted as *V*_MR_ = {*v*_MR,*j*_}, *j =* 1,… *V*_1_. All are preoperatively segmented from the MR data, as shown in Figure 2b. The source feature *Z*_US_ is a set of manually-picked points, including a subset of point *K*_US_ = {*k*_US,*i*_}, *i =* 1,… *K*_2_ selected on kidney contours (including transverse and longitudinal contours) in all used US slices, and a subset of points *V*_US_ = {*v*_US,*i*_}, *i =* 1,… *V*_2_ on hilum vein and IVC surfaces in *U*_1_, *U*_2_. Because we only use two pairs of orthogonal slices, the point selection will be completed within reasonable time. The categorized feature data will aid the registration by avoiding local minima and reducing computation time. Let *ck*_*i*_ and *cv*_*i*_ be the closest points to *k*_US,*i*_ and *v*_US,*i*_, respectively. Then, the OICP registration can be estimated as

(3) $$\begin{array}{l} \;\\ T_{\mathrm{reg}}^{*}=arg\;min\left[\frac{1}{K_{2}}\sum_{i=1}^{K_{2}}||T_{\mathrm{reg}}k_{US,i}-ck||\right]+\frac{1}{V_{2}}\sum_{i=1}^{V_{2}}||T_{\mathrm{reg}}k_{US,i}-ck||\text{,} \end{array}$$

**Figure 4:**
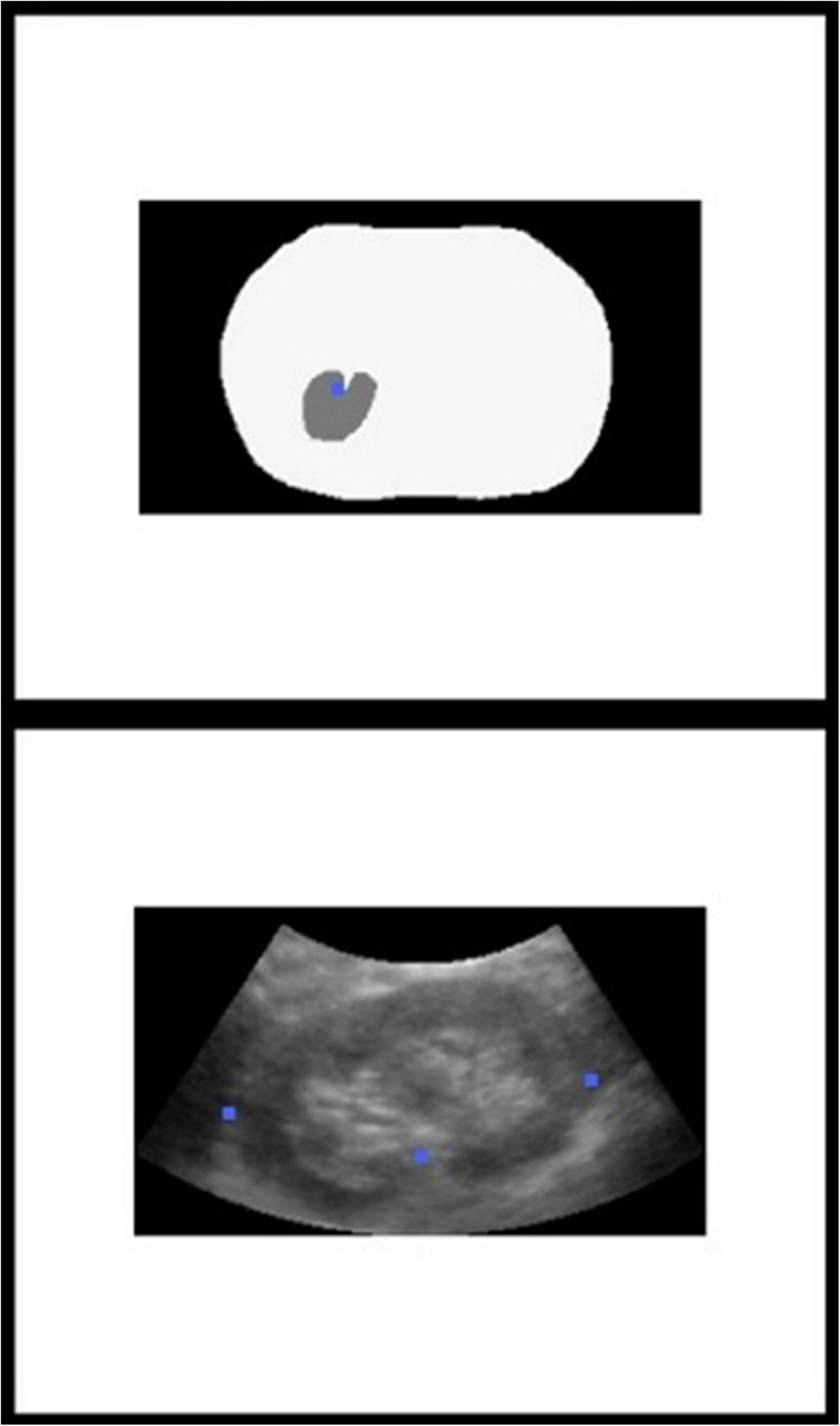
**The registration initialization.** Three pairs of landmark points are selected from the cranial end, caudal end and kidney hilum to initialize the registration.

The estimate starts with calculating an initiate transformation *T*_0_ by aligning three pairs of landmark points selected from the cranial end, caudal end and kidney hilum on both the US slices and the MR segmentation images, as shown in Figure 4. The initial alignment can be calculated using the Procrustes analysis [19]. This preprocessing allows quick algorithm convergence without falling into local minima. Assuming a fine initial registration, the following optimization can be constrained within a small translation range ± *A* and a small rotation angle range ± α, thus reducing the computing time while improving the reliability. The translation and rotation are then further optimized to minimize the mean square error (MSE). The entire optimization is summarized below.

**Input**: *K*_US_ and *V*_US_ manually picked from orthogonal US slices *U*_1_, *U*_2_, *U*_3_ and *U*_4_, *K*_MR_, *V*_MR_ preoperatively segmented from the MR volume;

**Output**: *T*_*R*_;

**Initiation**: The starting registration *T*_0_ is obtained by aligning the three pairs of landmark points.

**Iteration**: **for***n =* 1 to *n*_max_ or until convergence **do**

1. Compute the closest point $ck_{i}=C\left(T_{n-1}k_{\text{US},i},K_{\text{CT}}\right)$for *i =* 1,…, *K*_2_ and $cv_{i}=C\left(T_{n-1}v_{\text{US},i},V_{\text{CT}}\right)$ for *i =* 1,…, *V*_2_;

2. Compute an update *T*_*n*_ that minimizes the MSE between *T*_*n*-1_*Z*_US_ and {*ck*_*i*_}∪{*cv*_*i*_} with translation within ± *A* and rotation within ± *α*.

3. End the iteration when *n = n*_*max*_ or the decrease of the MSE is below a threshold *h*.

**Figure 5:**
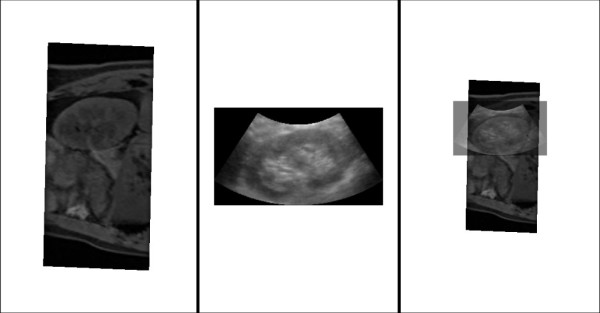
**The corresponding US and MR slices from a healthy volunteer.** Central: the US slice containing a complete kidney. Left: the corresponding MR slice cut from the MR volume. Right: overlay the US slice onto the corresponding MR slice.

With the final estimate of *T*_*R*_, the transformation from the US plane to the MR image space *S*_MR_ can be given by $T=T_{R}^{i}T_{T}T_{C}$. Figure 5 shows the corresponding US and MR slices from a healthy volunteer, where the longitudinal kidney contour is well aligned.

#### (iv). Augmented US-based guidance

**Figure 6:**
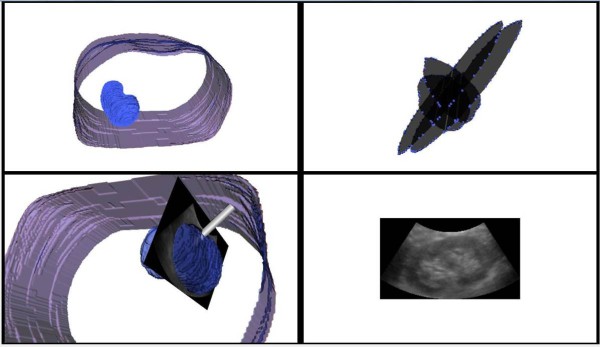
**The US-augmented PRA guidance interface.** Left top: preoperative MR models of kidney and skin. Right top: registration features extracted from orthogonal US slices. Right bottom: US slice. Left bottom: augmented US-based intraoperative guidance.

Based on *T*_*R*_, the planning and MR anatomical models can be transferred from the preoperative space *S*_MR_ into intraoperative space *S*_tra_. A puncture trestle is mounted to the US probe to restrict the needle trajectory to several given angles within the US plane. The needle position can be precisely measured by the tracker in real time and fused in the guidance image as a virtual model. The visual guidance is provided by augmenting the US images with 3D anatomical models, the planning and the virtual needle, as shown in Figure 6.

One should notice that the transformation *T*_*R*_ is calculated using only US slices at the maximum exhalation positions. Therefore, at the other stages of the respiratory circle, the accuracy of registered needle trajectory cannot be guaranteed because of the organ shift and soft-tissue deformation. In such a case, we expect to perform the puncture at maximum exhalation positions. To this end, we make use of the respiratory gating technique proposed above to plot the respiratory curve (as shown in Figure 3) in real time. The maximum exhalation positions can then be detected by visual inspection of the surgeon. Meanwhile, the needle is inserted rapidly into the intrarenal target. Under the presented image guidance, the puncture trajectory can be guided and guaranteed to be coincident with the planning.

### Experiments and evaluations

The proposed image guidance framework is evaluated in two stages: (i) evaluation of the registration performance in terms of the accuracy, precision and processing time measured on human data, (ii) evaluation in terms of puncture accuracy and perceptual quality assessed by four urologists on kidney phantom trials.

#### (i). Registration evaluation

Here, we aim to measure the registration accuracy in terms of the root mean square (RMS) target registration error (TRE) on MR and US data provided by volunteers. Because no gold standard is available, the MR to densely-sampled US volume registration proposed in [17] was used as a bronze standard [20].

The true-FISP MR data was acquired by scanning four healthy volunteers (distinguished by A, B, C, and D) on a Siemens MAGNETOM Trio Tim 3.0 T machine. Written informed consent from all volunteers was obtained. The voxel resolution was set to 1.12 × 1.12 × 1.00 mm^3^ to approximate the isotropy. The US images were acquired using a Mindray DC-7 machine with a 3.5 MHz abdominal probe and then captured using an EDIUX NX video grabber from CANOPUS. A passive Polaris system from Northern Digital Incorporation (NDI) was used for position tracking. The US probes was mounted with optically-tracked reflective markers such that the position of the probe can be tracked. The Mindray US machine provides a built-in calibration application to output the distance from the left bottom of the US slice to the center of the arc-shaped probe surface, such that the transformation *T*_*C*_ can be calculated directly. Note that for each imaging depth, the built-in US calibration algorithm only need to run once before the surgery. Then, the output can be stored in a mapping table between transformation *T*_*C*_ and imaging depth for future use. The transformation *T*_*T*_ was calculated automatically by means of the tracker’s real time output. Given *T*_*C*_ and *T*_*T*_, all US slices can be calibrated and located in the tracker space *S*_tra_, as described in section 2.

For acquiring one desired US slice at the maximum exhalation via the proposed respiratory gating, 50 to 100 slices were acquired at a stable rate of 20 slices per second using the video grabber. For calculating the bronze standard registration, 125 US images were selected at the maximum exhalation positions from each volunteer, covering from transverse to longitudinal views of the kidney. Four urologists with expertise in US-guided renal intervention were asked to individually conduct the proposed registrations and the bronze standard registration for each volunteer (denoted as Test 1–4). For calculating the OICP registration, the translation range and the rotation range are set to *A =* 28 mm and *α =* 30°, respectively. The threshold *h* for terminating the iterations was set to 0.1.

The accuracy can then be measured as follows. All voxel positions within the kidney model were transformed from *S*_MR_ into *S*_tra_, using both the proposed registration method and the bronze standard. The RMS error between the two corresponding position sets was then calculated. Thus, the accuracy in terms of RMS TRE was obtained. To evaluate the precision, or repeatability of the proposed method, the RMS distance from the transformed positions to their average, i.e., the standard deviation, was calculated. The processing time for the proposed registration was also recorded, including the Procrustes initial registration and OICP optimization.

#### (ii). Phantom trials

A triple-modality (CT, MR, US) abdominal phantom model 057 from Computerized Imaging Reference Systems (CIRS) was used for the phantom trials. The internal structure of the model 057 includes partial abdominal aorta, partial vena cava, spine and two partial kidneys each with a lesion. The lesions are high contrast relative to the background in MR and can be barely identified in US. Each lesion can be punctured 3–5 times. These features make it a useful tool for evaluating the targeting accuracy of a multimodality image-guided PRA.

First, the phantom was scanned with the same Simons MR machine. The MR volume data was then pulled onto the augmented-US based guidance system for surgery planning. With the 3D reconstruction of the segmented kidney, lesions, spine and skin, two planning trajectories were defined, each including an entry point on the skin and a target point within a lesion.

**Figure 7:**
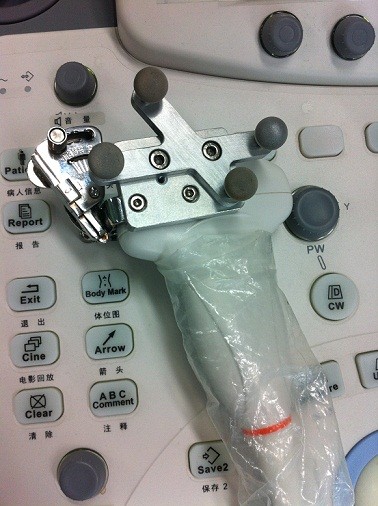
Ultrasound probe mounted with the optically-tracked markers and the puncture trestle.

**Figure 8:**
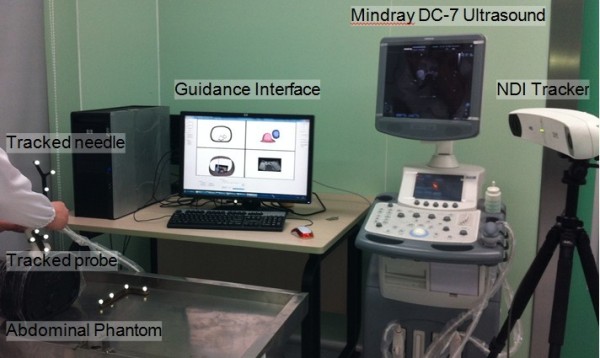
The image-guided intervention trial on an abdominal phantom.

The same four interventionists were asked to perform the RFA on the phantom (Test 1–4). Two pairs of orthogonal US slices containing longitudinal and transverse contours of the kidney were selected from real time US data for MR-US registration. Because the phantom is approximately rigid, the breathing gate technique was not used here. Three points from the cranial end, the middle, and the caudal end of the kidney were used for the initial alignment. 8 to 15 points from the kidney surface and the vena cava were used for the OICP registration. A rigid plastic needle equipped with four fixed optically-tracked markers was used. A puncture trestle was mounted to the US probe such that the needle trajectory was restricted, as shown in Figure 7. This needle can be located in real time in space *S*_tra_ and was intended to reach the target lesion. By observing the proposed visualized guidance, each interventionist performed two needle punctures. The environment of the phantom test is shown in Figure 8. After each PRA trial, MR scanning was performed to assess the puncture accuracy. The distance between the needle tip and the lesion center, denoted as needle-target distance (NTD), was measured based on the multiplanar reconstructed images.

The perceptual quality of the image guidance system for surgeons is very important, as their satisfaction relates to the therapeutic impact in selecting the system in clinical practice. The perceptual quality for the proposed PRA was rated in terms of three criteria according to a custom scoring system, as follows:

Intervention Improvement from 5 to 1 respectively denotes significant, meaningful, moderate, fair, and little localization improvement when the proposed guidance is employed. Workflow Impact indicates the acceptability of the proposed workflow, where 5 to 1 respectively denote positive, acceptable, acceptable after training, acceptable with reluctance, and unacceptable. Clinical Relevance denotes the clinical value of the proposed framework, where scores 5 to 1 correspond to values of high, moderately high, medium, moderately low to low. The evaluators were allowed to rate *x*.5 that represents an assessment between *x* and *x +* 1.

## Results and discussions

### Registration evaluation

All results are given in Table 1 and Table 2. The results in Table 1 show a good convergence to the bronze registration with a mean RMS TRE of 3.53 mm. We believe the proposed approach has benefited from the orthogonal geometry of the used US slices, where local minima can be avoided. A positive factor is the fine initiate registration conducted by the expert urologists.

**Table 1:** Measurement of RMS TRE accuracy (in millimeters) and processing time (in minutes plus seconds)

**Volunteers**	**Criteria**	**Test 1**	**Test 2**	**Test 3**	**Test 4**	**Mean**
A	RMS TRE	2.29	1.37	2.95	2.70	2.33
	time	3^′^22^″^	10^′^10^″^	7^′^16^″^	9^′^02^″^	7^′^28^″^
B	RMS TRE	3.73	3.11	4.10	3.27	3.55
	time	5^′^15^″^	8^′^52^″^	5^′^21^″^	6^′^41^″^	6^′^32^″^
C	RMS TRE	3.32	2.93	3.06	3.44	3.19
	time	3^′^15^″^	8^′^36^″^	5^′^22^″^	7^′^15^″^	6^′^7^″^
D	RMS TRE	4.52	5.24	4.72	5.76	5.06
	time	5^′^42^″^	8^′^17^″^	6^′^38^″^	8^′^26^″^	7^′^16^″^

**Table 2:** Measurement of the registration standard deviation (in millimeters)

**Volunteers**	**A**	**B**	**C**	**D**	**Mean**
SD	0.77	0.51	0.81	1.16	0.81

A previous study presented an intensity-based registration of CT and three-dimensional (3D) US kidney images and the average error was 5.36 mm [10]. The bronze standard and accuracy criteria used were similar, so the comparison with our method makes sense. The different results can be explained by the following. This previous work used correlation ratio (CR) between the intensity CT images and the gradient US as similarity measure. We believe the correlation between CT and US is not that explicit and the accuracy will be influenced. On the other hand, this previous work did not take the breathing motion into account. In another prior study on computer-assisted access to the kidney, kidney surface-based registration was used to align CT and freehand 3DUS [21]. They did not report the RMS error but found failed examples. A prior work in [11] evaluated the accuracy and precision of their registration between MR and freehand 3DUS for human livers based on the vessel probability images. They used similar bronze standard and accuracy criteria to assess the registration performance. The RMS TRE was reported to be 3.6 mm with a relatively high mean failure rate of 5.6%. Furthermore, all these methods in [10,11,21] need to acquire relatively dense US slices intraoperatively. It is difficult to be accepted in clinical use. This problem may be solved by using the real-time 3DUS probes instead of the freehand method [22].

In the study in [9], the spatial accuracy of the commercial registration technique VNav was examined via clinical trials of radiofrequency ablation of liver tumors. The max difference (denoted as Dmax) between the target lesions in both aligned CT and US was measure. The mean Dmax was 6.55 mm when CT was performed immediately before VNav. The authors claimed that it was considered to be clinically accepted for liver tumor ablation. However, a small Dmax meant a small distance between the aligned US and CT but not an accurate registration, because the gold standard of the registration was not available. No literatures on the accuracy of VNav in kidney intervention were found. We believe it is a reasonable hypothesis that our method would be superior because the registration landmarks for VNav are insufficient and less efficient in selection, especially in the US images of kidney.

The results in Table 1 imply that for different data the proposed registration is rather robust. Specifically, for different surgeons and volunteers (presenting variability of pose initiation, landmark identification and interindividual anatomy) the registration accuracy does not change largely. In other words, no evident outliers were found. Table 1 also shows the operation can be completed within a reasonable time (on average 6'4"). Note that the bronze standard registration took about 37 minutes on average. The better accuracy and less operating time validate the high efficiency of the orthogonal slices in MR to US registration of the kidney. It should be pointed out that the processing time for intensity-based registration is less due to its property of automatic calculation without manual feature extraction. For example, the prior work in [10] reported a registration time of 80s. Note that this time did not count the freehand 3DUS data acquisition duration.

Although there is no evident outlier, the results indicate that the registration accuracy is to some extent affected by the volunteers. It is mainly because of the fact that the anatomical features of kidney, hilum vessels, and IVC vary between people, which will lead to different US imaging quality. Similar findings have been reported by others [21]. For example, it is difficult to clearly identify kidney contours and vessels in US images of obese people. That is exactly why the registration for volunteer D was less accurate. It suggests that a patient selection may be required for optimal outcomes. On the other hand, the duration of operation exhibits an observable dependence on the surgeons. For example, among all the tests, the elapsed time for the first surgeon is the shortest while that for the second surgeon is relatively long. Note that the registration feature point selection is the most time-consuming procedure.

The results in Table 2 show that the registration is repeatable for the tested volunteers. In other words, the results are not significantly related to the surgeons performing the registration. A reasonable explanation is that the orthogonal geometry of the US slices reduces the random fluctuation in the initial landmark selection and the feature point selection. All these results in Table 1 and Table 2 suggest that the hilum vessels, IVC and kidney contour together can be used as preferred landmarks.

Note that the above evaluation method cannot detect the presence of errors induced by the segmentation and calibration. Considering the pixel per millimeter ratio and the physical size of the MR image, it is reasonable to conclude a less than 1.6 mm (or 1.5 pixels) error for the segmentation. The typical error for the calibration method used is less than 1 mm. Note that the experimental results cannot account for the system error for both tested registration methods.

### Phantom trials

The results of the accuracy in terms of NTD are shown in Table 3. The mean NTD over the four tests was 2.08 mm for the left lesion and 1.85 mm for the right one. It can be seen that the distance was smaller than the registration error measured on human data. One major reason is that the phantom is a rigid body with clear registration features. The mean duration for all phantom PRA tests was 4'26". It implies that the accurate RFA can be finished within acceptable time. The difference between the four tests was relatively small, indicating a robust registration for different operators.

**Table 3:** Measurement of the NTD (in millimetres) and the operating time (in minutes plus seconds) for the kidney phantom test

**Target**	**Criteria**	**Test 1**	**Test 2**	**Test 3**	**Test 4**	**Mean**
Left lesion	NTD	2.9	1.6	2.1	1.7	2.08
	time	5^′^15^″^	3^′^40^″^	3^′^56^″^	5^′^02^″^	4^′^28^″^
Right lesion	NTD	2.2	1.7	1.9	1.6	1.85
	time	4^′^25^″^	4^′^32^″^	3^′^1^″^	5^′^41^″^	4^′^24^″^

The previous phantom study on kidney intervention in [21] reported a NTD of 4.7 mm. Our method achieved a better accuracy with less US slices used. The previous study claimed that the main inaccuracy came from symmetrical shape of the phantom which introduced potential indeterminations and from the needle deformation during puncture. In our test, a puncture trestle was used to restrict the needle trajectory. This may reduce the needle deformation, at least to some extent. In previous study in [5], the accuracy of the VNav was evaluated via puncture tests on a customized phantom. The mean NTD was 2.7 mm for the trials performed by an experienced radiologist while 3.1 mm for a medical resident without experience. As the phantom is a rigid body with clear and fixed anatomical landmarks, the accuracy difference on phantom tests for VNav and our proposed method is not significant.

Furthermore, real-time 3D US has shown a promising future in guiding PRA procedure. In the pioneer study in [23], the authors determined the feasibility and accuracy of 3D US reconstruction of the pelvicaliceal system (PCS) in an in vitro porcine kidney. A further study in [24] has demonstrated that 3D US is more accurate than 2D US for PCS measurements on a customized phantom. The authors in [24] then performed 3D US-guided punctures on that phantom and proved the feasibility. Therefore, it is a nature idea that the accuracy and repeatability would be improved further by intruding a 3D US system into our guidance framework. Moreover, to fuse the 3D US images with preoperative planning such as CT pyelography [25] and MR urography [26] should be more accurate and intuitive for guiding PCNL. On the other hand, in the case of percutaneous renal ablation where the lesion is invisible in US images, image fusion of 3D US and CT/MR planning should be a more accurate solution compared with the use of 2D US. To this end, further evaluation on in vivo human kidneys should be conducted.

The scoring results shown in Table 4 imply that they appreciated the use of the proposed image guidance tool in renal intervention due to the accuracy improvement and especially the intuitive 3D guidance, although maybe special training is needed. All surgeons agreed that careful setups including optimal planning, landmark selection and patient selection were required for satisfactory outcomes. One surgeon suggested improving the respiratory gating technique, as the respiratory tracking is less sensitive when the US probe is placed along the midaxillary line. It was also pointed out that one impediment in delivering the presented guidance system in clinical practice is the distance between the preoperative planning workstation (often located at the doctor’s room) and the operating room. In such a case, hospital network that efficiently transfers planning data between the planning workstation and the OR is required.

**Table 4:** Assessment of the perceptual quality on a kidney phantom

**Criteria**	**Test 1**	**Test 2**	**Test 3**	**Test 4**	**Mean**
Intervention Improvement	4	3.5	4.5	4	4.0
Workflow Impact	3.5	3	3.5	3	3.3
Clinical Relevance	4	4	4	3.5	3.9

## Conclusions

The presented image guidance is feasible and promising for PRA procedure. With careful setup, it can be efficient for overcoming the limitation of current US-guided PRA by providing accurate and robust target localization abilities, intuitive guidance interface and satisfactory perceptual quality.

## Competing interests

The authors declare that they have no competing interests.

## Authors' contributions

ZCL and KL contributed equally to this work. They together implemented the presented image guided framework, conducted the experimental study, and were responsible for the for the data analysis. HLZ participated in the designing of the experiment, performed the registration test and phantom trials. KC implemented the registration algorithm and the image reconstruction algorithm. JG participated in the registration algorithm design and experimental data analysis. LW provided the experiment infrastructure and contributed to the result discussion. All authors read and approved the final manuscript.
